# Supplementary material for: The topology of genome-scale metabolic reconstructions unravels independent modules and high network flexibility
Source: PLoS Comput Biol. 2022 Jun 27;18(6):e1010203. doi: 10.1371/journal.pcbi.1010203 (PMC9269948; doi:10.1371/journal.pcbi.1010203)
Supplement: S1 Text — A: Checking model capabilities after removing antiporter reactions (in S1 Text). B: Calculation of correlation in gene expression between structurally reversible reactions of distance 2 (in S1 Text).: Missing Infeasible Patterns (in S1 Text). D: Study of directionality correlation between structural reversible reactions (in S1 Text). (DOCX) [file pcbi.1010203.s001.docx]

**Title:** The topology of genome-scale metabolic reconstructions unravels independent modules and high network flexibility

**Short Title:** Identification of metabolic modules based on model topology

Verónica S. Martínez ^1,2#^, Pedro A. Saa^3,4#^, Jason Jooste^1^, Kanupriya Tiwari^1^, Lake-Ee Quek^1,5^, Lars K. Nielsen^1,2,6,7*^

^1^ Australian Institute for Bioengineering and Nanotechnology (AIBN), The University of Queensland, Brisbane, Queensland, Australia

^2^ ARC Training Centre for Biopharmaceutical Innovation (CBI), Australian Institute for Bioengineering and Nanotechnology (AIBN), The University of Queensland, Brisbane, Queensland, Australia

^3^ Departamento de Ingeniería Química y Bioprocesos, Escuela de Ingeniería, Pontificia Universidad Católica de Chile, Santiago, Chile

^4^ Instituto de Ingeniería Matemática y Computacional, Pontificia Universidad Católica de Chile, Santiago, Chile

^5^ The Charles Perkins Centre, School of Mathematics and Statistics, The University of Sydney, Sydney, NSW, Australia

^6^Metabolomics Australia, The University of Queensland, Brisbane, Queensland, Australia.

^7^ The Novo Nordisk Foundation Center for Biosustainability, Technical University of Denmark, Kgs. Lyngby, Denmark

^#^ contributed equally

**Supporting Information**

**Supporting text A: Checking model capabilities after removing antiporter reactions**

In order to check that the overall model capabilities of Recon 2.2 model were not disrupted by the removal of antiporter reactions FVA was run (Table E in S1 Tables). Most reactions kept the same range of flux. Only 12 reactions became blocked, but the metabolites that those reactions were transporting were initially only part of transport reactions and were no present in any other metabolic reaction. Eleven transport reaction became irreversible, but for all but 3 metabolites there were other transport reactions to transport the metabolites in the opposite direction. The three metabolites that in the reduced model where only being transporter in one direction, the opposite transport direction in the full model was only part of a loop. Only one reaction (R_CLI2tex) kept the same reversibility but the maximum flux was reduced by half, but this reduction in flux did not affect the flux to any other reaction of the model. Most importantly, in general the reduced model was able to produce and consume the same metabolites after the reactions removal, as well as kept the maximum specific growth rate.

**Supporting text B: Calculation of correlation in gene expression between structurally reversible reactions of distance 2.**

The code used to calculate the correlations and plot the data is available (Correlations.zip file). For the *E.coli* iJO1366 model, we downloaded the PRECISE database [1], which is read by the matlab script, *GenesCorrelations.m* (S1 Data). For the Human Recon 2.2 model, we downloaded human expression data from the GTEx (The Genotype-Tissue Expression) project [2] (<https://gtexportal.org/home/datasets>, Version V8). The TPM file is too large to be read using matlab directly and we pre-processed the data using python to estimate the correlation between genes and standard deviation of transcriptomic data. The script *filter_by_vero_ids_and_corr.py* was used to filter the GTEx TPM counts table and compute the gene-gene correlation tables with and without log2(TPM + 1) transformation (S2 Data). More information on the script is available in the *filter_and_corr.pbspro* file. The data from files *Gtex_vero_genes_10tpm_corr.csv* and *Gtex_vero_genes_10tpm_STD.tsv were subsequently* analyzed using matlab script *GenesCorrelationRecon22.m* to generate the plot of distribution of correlation and estimate the statistics. The correlation estimations can be found in Tables D and G in S1 Tables.

**Supporting text C: Missing Infeasible Patterns**

Some infeasible conditions were not directly identified as a consequence of mass balance or loopless condition rules. The initially identified infeasible patterns (due to mass balance and loopless condition rules) were used as a starting point to find the missing patterns. These missing infeasible patterns were similar to the already identified patterns. A two steps algorithm was used:

1. *Search of infeasible patterns not identified by rules*. Using the identified infeasible patterns that contain 3 or more fixed directions, one constraint was removed at a time and replaced by constraining another initially unconstrained reversible reaction. The new potential infeasible pattern is checked for feasibility, if it is found infeasible it is added to the list of infeasible patterns. When this search is finished, for the repeated patterns only one copy is kept on the list, and long patterns that are contained on smaller ones are removed from the list.
2. *Reduction of the newly identified infeasible patterns*. The constraints of the newly identified infeasible patterns are removed one by one and a feasibility check is performed. This process is performed until finding the core constrains that makes the pattern infeasible. It is possible that when the new patterns are reduced, actually the constraints that made the patterns infeasible are the same. Therefore, a check of repeated patterns in done and only one copy of the pattern is kept on the list.

**Supporting text D: Study of directionality correlation between structural reversible reactions**

To further understand the coupling between structural reversible reactions, we evaluated the directionality correlation between structural reversible reactions. Module 15 was studied because is the largest and more complex of the modules. We note, however, that other smaller module showed similar correlation behavior (Table M in S1 Tables). Given the extremely large number of DTs in module 15 (> 4.8x10^10^), storage of all feasible DTs was infeasible which precluded the computation of the correlation between the structural reversible reaction directionalities. To overcome this obstacle, a random sample of 1x10^5^ representative DTs was instead generated and analyzed for this purpose. For this task, the loopless Artificially Cantered Hit-and-Run on a Box (ll-ACHRB) sampler [3] was employed to randomly generate mass-balanced loopless flux distributions in the interior of the feasible flux space. Briefly, ll-ACHRB was run until 10^5^ unique DTs were obtained, which amounted to run the sampler for approximately 5*10^6^ samples. This sample was then employed for the subsequent analysis.

Our analysis revealed a very poor correlation in the structural reversible reactions of the DTs sample (Fig I A in S1 Fig). The absolute correlation distribution displayed a median of 0.011 and, and a 5% and 95% percentile of 0 and 0.119, respectively. These results suggest a weak coupling between structural reversible reactions, which points to a high topological flexibility in the studied module. We further investigated the presence of clusters of highly coupled reversible reactions within module 15 by enumerating the largest sets of fully connected reactions (maximal cliques) (see [4] for more details). Here, connected reactions were defined as those with an absolute correlation higher than a defined cut-off, r_cutoff_. As expected, the higher the value of r_cutoff_, the least and smaller the size of the cliques (Fig I B and C in S1 Fig). Notably, there were only five cliques made of reactions with a high correlation (> 0.85). Inspection of these cliques revealed they were all composed of only 2 reactions each, involving an exchange and an internal reaction consuming the sourced metabolite. This type of coupling is not unexpected as exchange reactions commonly exert massive coupling and blocking of reactions at the boundary of metabolic networks [5]. Overall, these results confirm that not only structural reversible reactions from different modules are independent from each other, but they are also largely independent from other structural reversible reactions from the same module. This confers a high flexibility to the structural reversible reactions modules, and more importantly, it likely confers high robustness to the network as a whole.

**References**

1. Sastry AV, Gao Y, Szubin R, Hefner Y, Xu S, Kim D, et al. The Escherichia coli transcriptome mostly consists of independently regulated modules. Nat Commun. 2019;10(1):5536. Epub 2019/12/05. doi: 10.1038/s41467-019-13483-w. PubMed PMID: 31797920; PubMed Central PMCID: PMCPMC6892915.

2. Carithers LJ, Ardlie K, Barcus M, Branton PA, Britton A, Buia SA, et al. A Novel Approach to High-Quality Postmortem Tissue Procurement: The GTEx Project. Biopreserv Biobank. 2015;13(5):311-9. Epub 2015/10/21. doi: 10.1089/bio.2015.0032. PubMed PMID: 26484571; PubMed Central PMCID: PMCPMC4675181.

3. Saa PA, Nielsen LK. ll-ACHRB: a scalable algorithm for sampling the feasible solution space of metabolic networks. Bioinformatics. 2016;32(15):2330-7. Epub 2016/05/07. doi: 10.1093/bioinformatics/btw132. PubMed PMID: 27153696.

4. Gomes de Oliveira Dal'Molin C, Quek LE, Saa PA, Nielsen LK. A multi-tissue genome-scale metabolic modeling framework for the analysis of whole plant systems. Front Plant Sci. 2015;6:4. Epub 2015/02/07. doi: 10.3389/fpls.2015.00004. PubMed PMID: 25657653; PubMed Central PMCID: PMCPMC4302846.

5. Burgard AP, Nikolaev EV, Schilling CH, Maranas CD. Flux coupling analysis of genome-scale metabolic network reconstructions. Genome Research. 2004;14(2):301-12. doi: Doi 10.1101/Gr.1926504. PubMed PMID: ISI:000188811800012.
