# Supplementary material for: The topology of genome-scale metabolic reconstructions unravels independent modules and high network flexibility
Source: PLoS Comput Biol. 2022 Jun 27;18(6):e1010203. doi: 10.1371/journal.pcbi.1010203 (PMC9269948; doi:10.1371/journal.pcbi.1010203)
Supplement: S1 Fig — A: Characterization of Recon 2.2 modules dimensions. Almost half of the generated modules contain one reversible reaction, while 6% of the modules contain 10 or more reversible reactions (in S1 Fig). B: Infeasible flux patters of module 15 due to loopless rules. 5 of the 11 infeasible flux patterns are presented, each with a different colour. The arrows represent the reactions in the module (both reversible and irreversible) and the nodes the metabolites (in S1 Fig). C: Infeasible flux patters of module 15 due to loopless rules. 4 of the 11 infeasible flux patterns are presented, each with a different colour. The arrows represent the reactions in the module (both reversible and irreversible) and the nodes the metabolites (in S1 Fig). D: Infeasible flux patters of module 15 due to loopless rules. 2 of the 11 infeasible flux patterns are presented, each with a different colour. The arrows represent the reactions in the module (both reversible and irreversible) and the nodes the metabolites (in S1 Fig). E: Infeasible flux patters of module 15 due to mass balance rules. 14 of the 15 infeasible flux patterns are presented, each with a different colour. The arrows represent the reactions in the module (both reversible and irreversible) and the nodes the metabolites (in S1 Fig). F: Infeasible flux patters of module 15 due to mass balance rules. 1 of the 15 infeasible flux patterns are presented, each with a different colour. The arrows represent the reactions in the module (both reversible and irreversible) and the nodes the metabolites (in S1 Fig). G: Infeasible flux pattern of module 15 due to two metabolites mixed mass balance rules. The infeasible pattern is highlighted using red arrows. The arrows represent the reactions in the module (both reversible and irreversible) and the nodes the metabolites (in S1 Fig). H: Infeasible flux pattern of module 15 found using the heuristic algorithm. The two infeasible patterns are highlighted using red and blue arrows. The arrows [file pcbi.1010203.s002.docx]

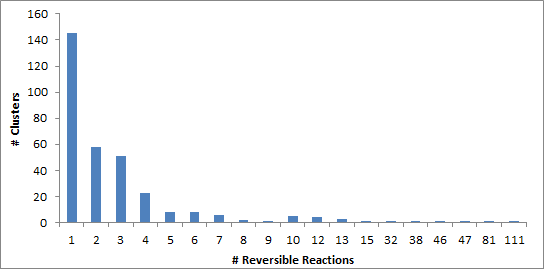


**Fig A: Characterization of Recon 2.2 modules dimensions.** Almost half of the generated modules contain one reversible reaction, while 6% of the modules contain 10 or more reversible reactions.


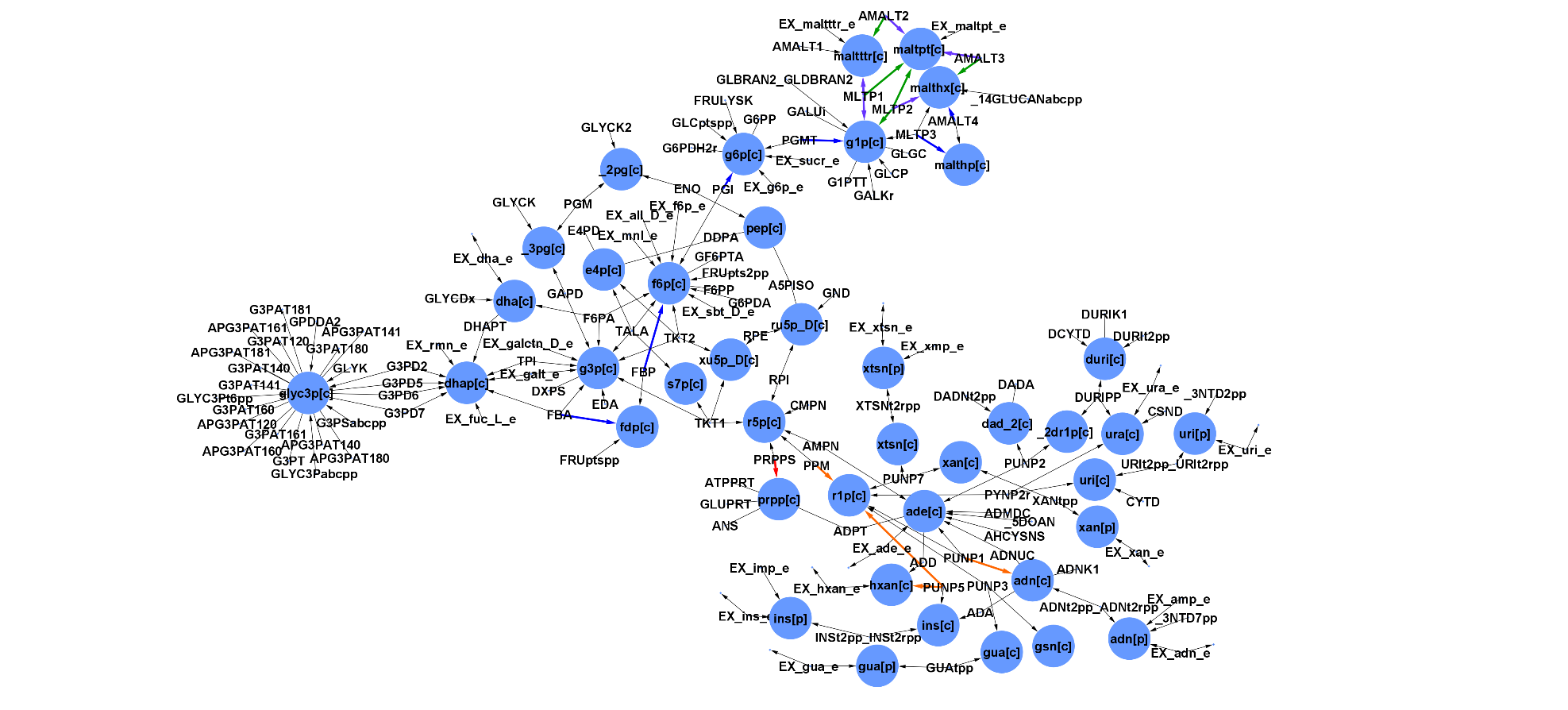


**Fig B: Infeasible flux patters of module 15 due to loopless rules.** 5 of the 11 infeasible flux patterns are presented, each with a different colour. The arrows represent the reactions in the module (both reversible and irreversible) and the nodes the metabolites.


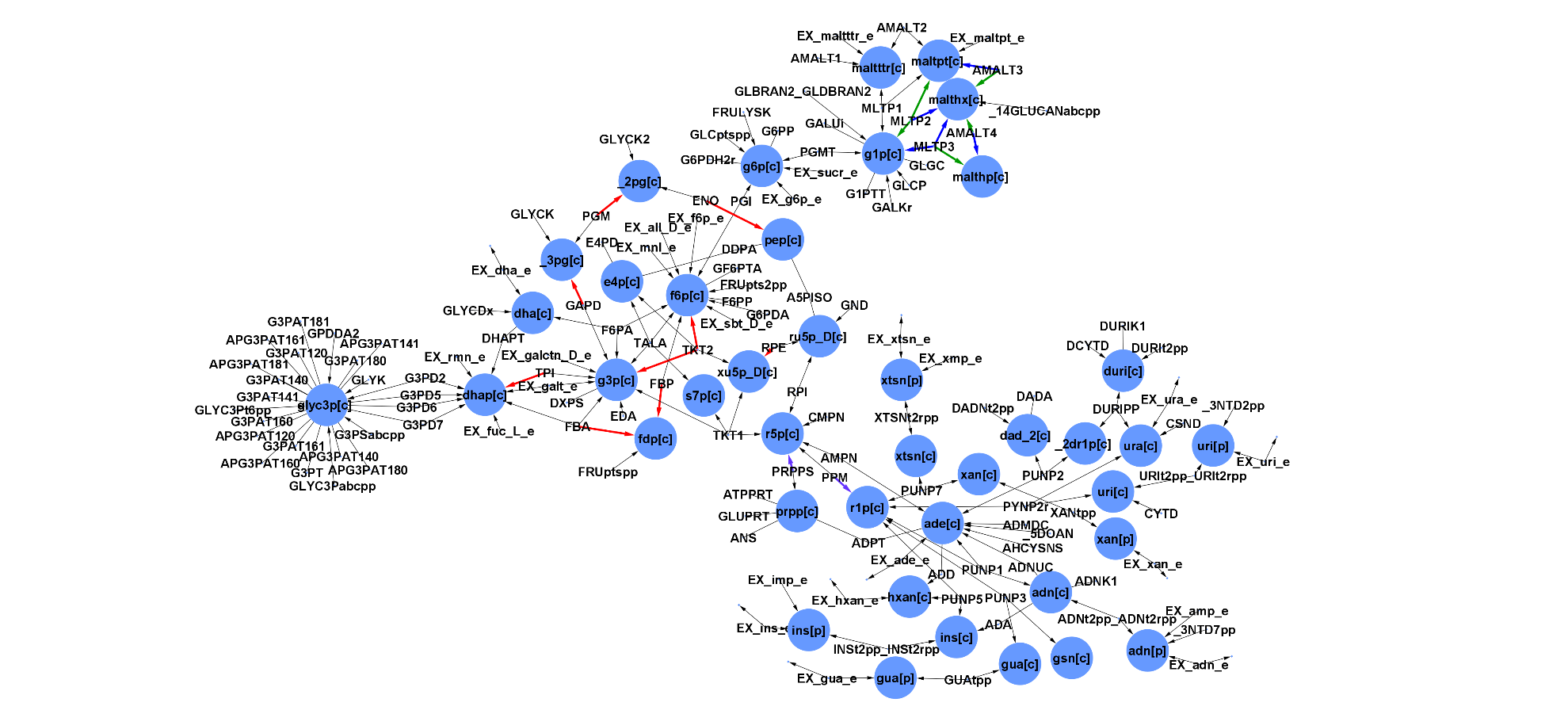


**Fig C: Infeasible flux patters of module 15 due to loopless rules.** 4 of the 11 infeasible flux patterns are presented, each with a different colour. The arrows represent the reactions in the module (both reversible and irreversible) and the nodes the metabolites.


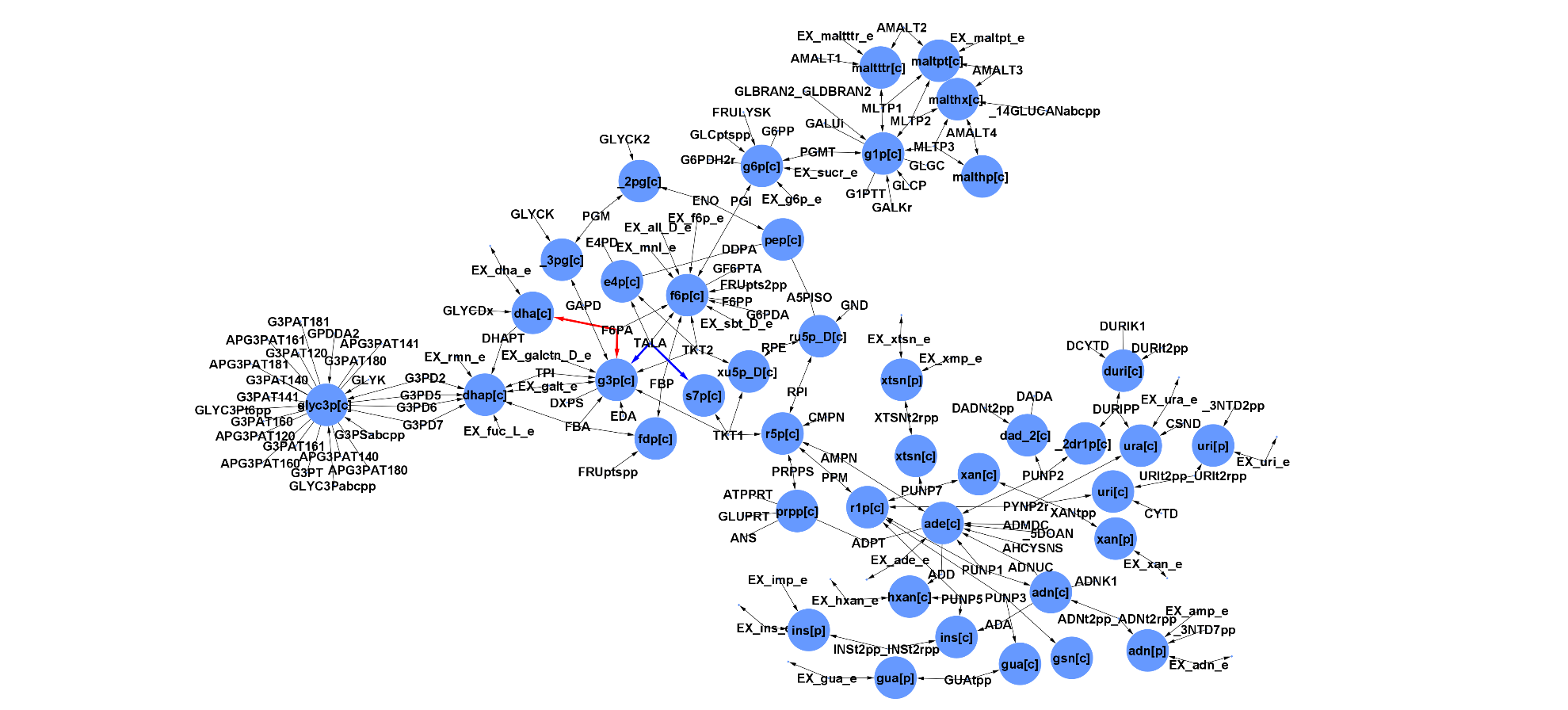


**Fig D: Infeasible flux patters of module 15 due to loopless rules.** 2 of the 11 infeasible flux patterns are presented, each with a different colour. The arrows represent the reactions in the module (both reversible and irreversible) and the nodes the metabolites.


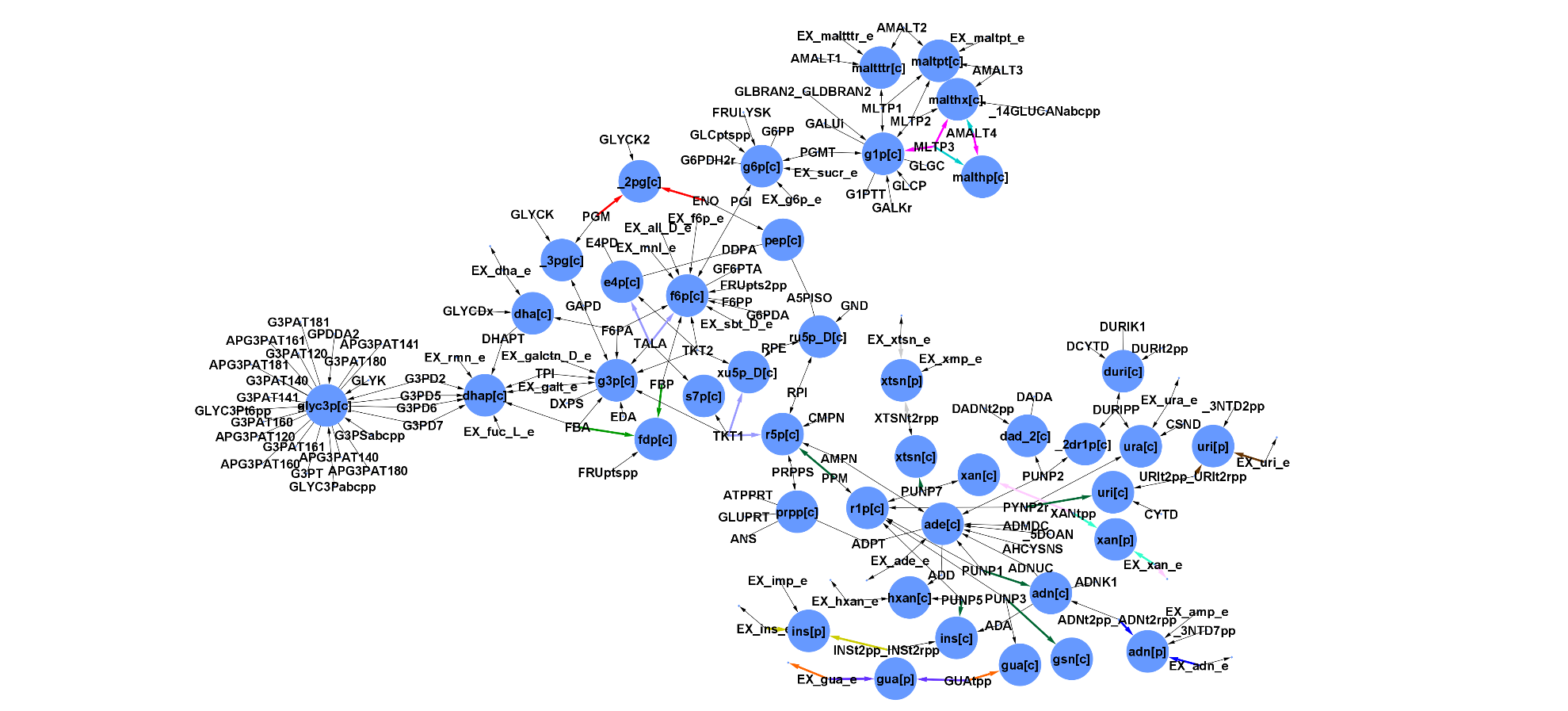


**Fig E: Infeasible flux patters of module 15 due to mass balance rules.** 14 of the 15 infeasible flux patterns are presented, each with a different colour. The arrows represent the reactions in the module (both reversible and irreversible) and the nodes the metabolites.


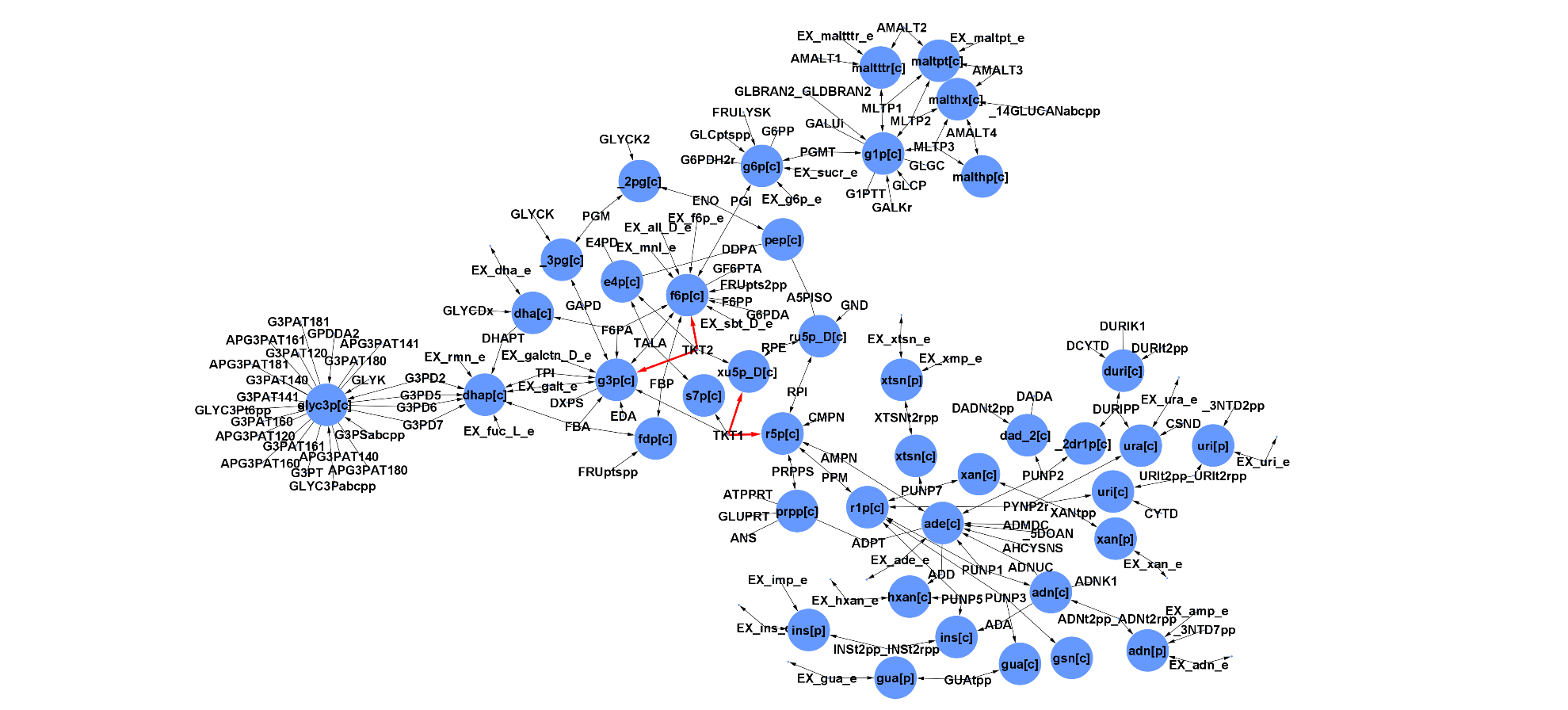


**Fig F: Infeasible flux patters of module 15 due to mass balance rules.** 1 of the 15 infeasible flux patterns are presented, each with a different colour. The arrows represent the reactions in the module (both reversible and irreversible) and the nodes the metabolites.


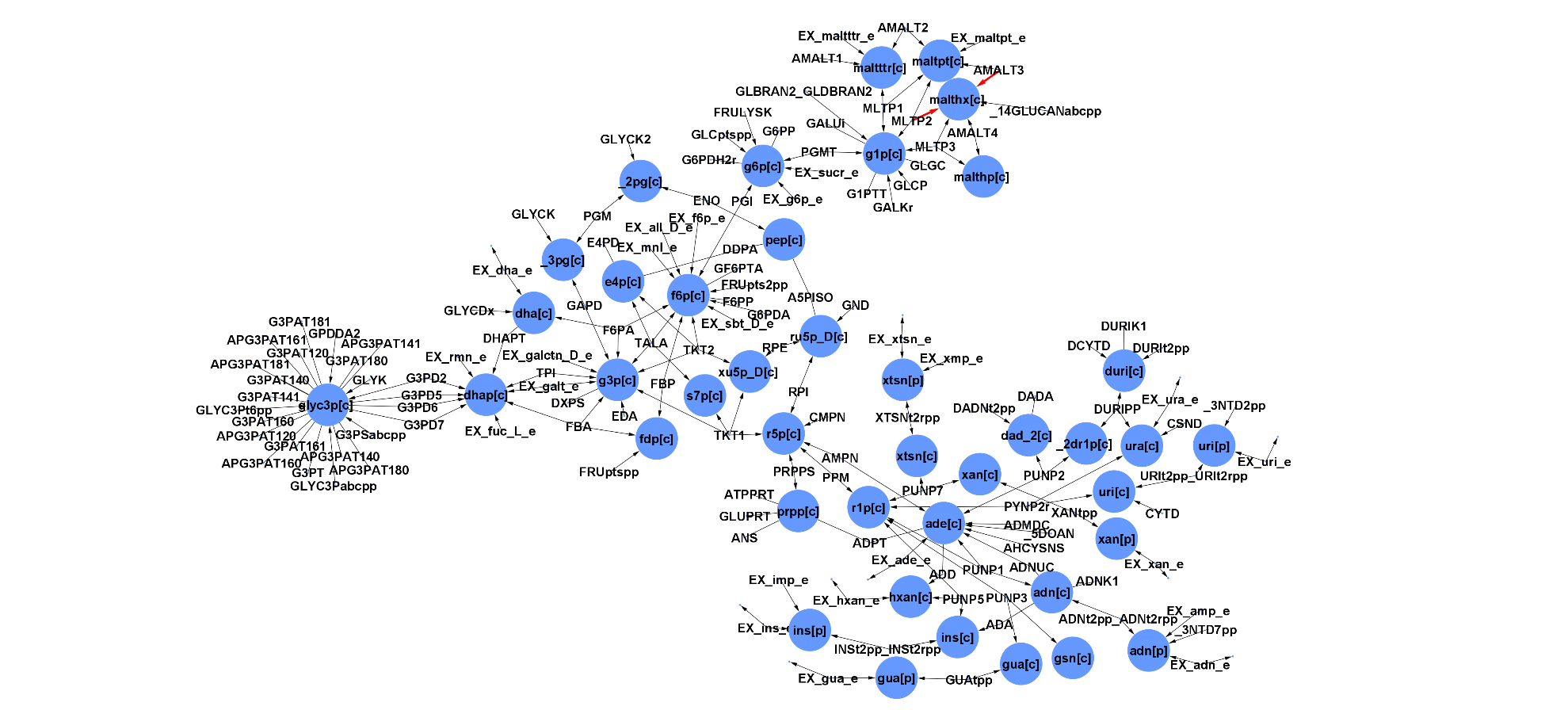


**Fig G: Infeasible flux pattern of module 15 due to two metabolites mixed mass balance rules.** The infeasible pattern is highlighted using red arrows. The arrows represent the reactions in the module (both reversible and irreversible) and the nodes the metabolites.


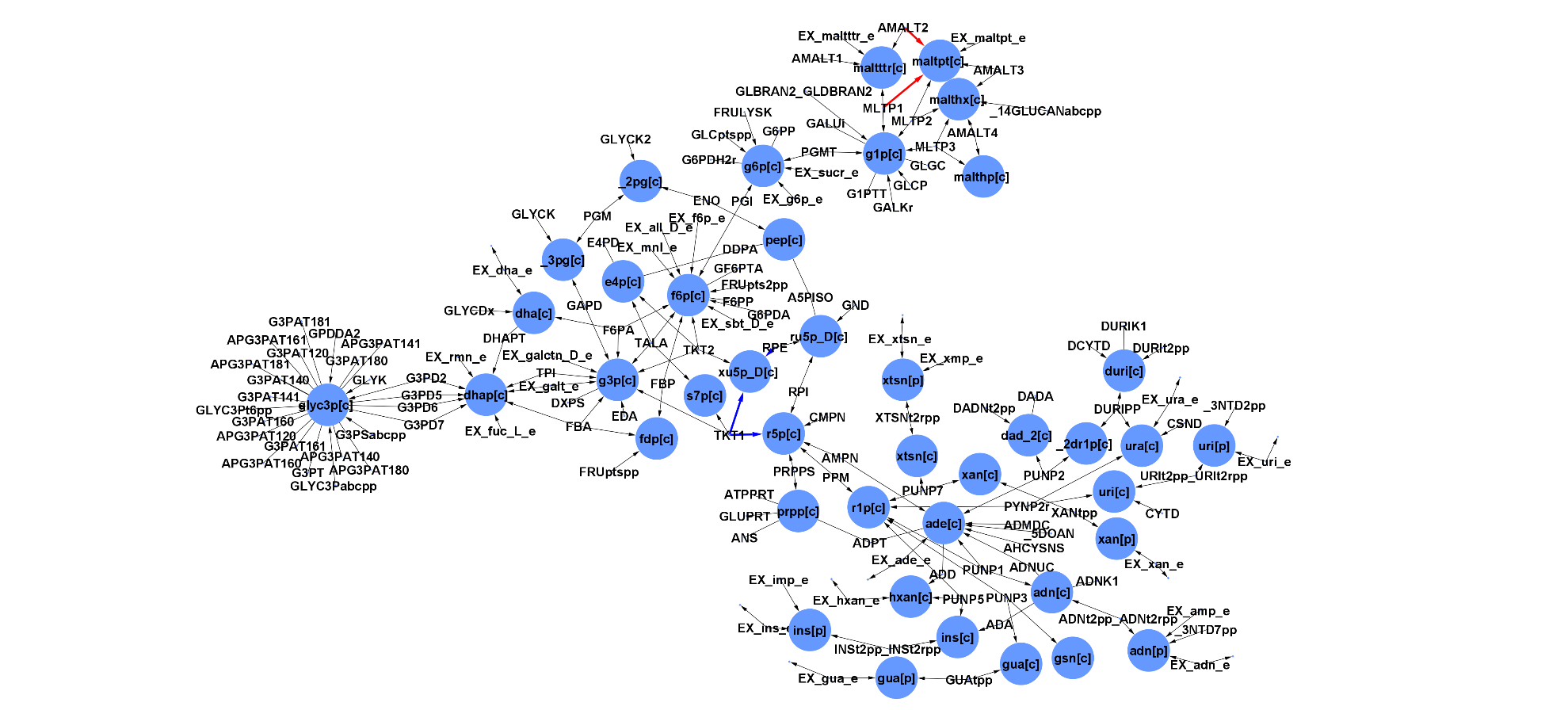


**Fig H: Infeasible flux pattern of module 15 found using the heuristic algorithm.** The two infeasible patterns are highlighted using red and blue arrows. The arrows represent the reactions in the module (both reversible and irreversible) and the nodes the metabolites.

**Fig I: Correlation analysis of the directionalities of the structural reversible reactions in module 15.** (A) Absolute correlation distribution of the directionalities in the DTs sample. (B) Cliques distribution and (C) Cliques size distribution for different absolute correlation thresholds. The higher the absolute correlation cut off, the least and smaller the size of the cliques found.
