# Supplementary material for: The topology of genome-scale metabolic reconstructions unravels independent modules and high network flexibility
Source: PLoS Comput Biol. 2022 Jun 27;18(6):e1010203. doi: 10.1371/journal.pcbi.1010203 (PMC9269948; doi:10.1371/journal.pcbi.1010203)
Supplement: Attachment — Submitted filename: Response letter 07_02_22.docx [file pcbi.1010203.s006.docx]

**Response to Editor and Reviewers' comments**

**PCOMPBIOL-D-21-01428**

**The topology of genome-scale metabolic reconstructions unravels independent modules and high network flexibility**

Thank you for your detailed review of our manuscript. We have addressed and considered the points and concerns raised by the reviewers. Below, we have addressed and answered the reviewers’ comments in a point-by-point response (in blue). Modifications to the main text are shown in red. In addition to the specific comments noted below, we have updated the manuscript's main text and updated its references. The list of references employed in this response can be found at the end of the letter.

**Reviewer #1**

We have read “The topology of genome scale reconstructions unravel independent metabolic modules and high model flexibility” by Martinez et al.  The authors explored the topology of metabolic network reconstructions by defining sets of thermodynamically isolated modules connected by irreversible reactions.  The modules corresponded with correlations in RNA transcription data.  A major focus was on Directed Topologies which were defined by fixing the direction of reversible reactions and then determining how many could carry flux.  The authors explored these topologies for metabolic networks of E. coli and Human metabolism.  Overall, the manuscript describes a nice approach for defining metabolic subsystems in an unbiased way.  However, we have several questions regarding the methods and assumptions used by the authors.  Unfortunately, the methods section lacking leading to several questions related to the robustness of the method.

**R:** We appreciate the positive comments of the reviewer about our methodology. Below, we have addressed the main concerns raised.

Major Comments:

R1.1. Our major concern relates to some ambiguity in the methods section.  The authors describe two steps of model reduction. The first uses FVA to estimate the flux of all network reactions.  However, the authors do not specify the constraints applied to their models prior to FVA.  For the E. coli model, were the FVA calculations run using default constraints (e.g. aerobic M9 minimal media + glucose?) or was this a fully open model?  What about for the human reconstruction?  Changing constraints, especially exchange reactions will change FVA results.  Thus, we must know what constraints were used.

**R:** Thank for raising this important point. We acknowledge that this information was missing from our original submission, and it is key for interpreting the results. We have made this information clear in the revised version. In both cases, the models were analyzed assuming growth under aerobic conditions with glucose as main carbon source (i.e., the default model exchange constraints in both cases). We have added this information in the revised manuscript Methods section and the constraints in the Supporting Tables S8 and S9.

“Prior to model reduction and compression, the models were modified for mass balance and thermodynamic calculation consistency [32, 33]. In short, reactions catalyzed by different enzymes in either direction were lumped together, the species HCO3, CO3, CO2, and H2CO3 were aggregated as CO2tot and H2O was added to the other side of the reaction. Additionally, the oxidized and reduced FAD of mitochondria were replaced by the oxidized and reduced FADenz to represent the enzyme-bound FAD cofactor. Finally, the models’ original directionality constraints were used, which describe aerobic growth with glucose as main carbon source (Supporting Tables S8 and S9)”.

Moreover, we have clarified that our methodology was tested for the identification of metabolic modules under specific growth conditions. This enabled us to evaluate the biological relevance of the identified modules based on the available expression data. We note that the proposed methodology can also be employed in a more prospective manner if desired, for example, imposing open constraints on the exchanges. We have incorporated a comment about this point in the Introduction.

(First line, last paragraph of the Introduction) “This work presents a novel approach to generate topologically independent metabolic modules exploiting the network topology and directionality constraints. The E.coli iJO1366 [4] and Recon 2.2 [7] GeMs were subdivided in topologically independent modules and evaluated for their biological relevance under specific growth conditions.”

R1.2. Do the authors anticipate dramatically different results from altering these constrains? Would the identified modules change dramatically? Is a sensitivity analysis needed?

**R:** We certainly expect different results (variation in the number of metabolic modules or increase in the size of the modules) as the number of exchange constraints are relaxed since more nutrients could be consumed from the media. In such case, the initial FVA reduction step would remove a smaller fraction of reactions unable to carry flux. This will increase the number of total reversible reactions, which should also increase the number of isolated metabolic modules and/or the size of the modules. However, the exact magnitude of the variation in the size and/or number of modules is hard to determine *a priori* due to the inherent combinatorial nature of the problem (refer to [3, 4] for the complexity analysis of related problems). While a sensitivity analysis could provide some insights about this magnitude change, the design of such (sensitivity) analysis is not trivial and will be ultimately hard to interpret (again, owing to the complexity of the problem). Therefore, we have opted for generating context-specific modules that can be correlated with expression data, highlighting their biological relevance. In Discussion there is a general comment about the usefulness of our methodology.

“The biological relevance of the identified modules was demonstrated through gene expression analysis, which showed that the correlation between of reversible reactions of distance 2 was significantly high between reactions within a cluster and low between reactions in separate clusters. The majority of correlated enzymes catalysing reactions within the same module are highly correlated (more than 0.8 absolute value correlation across 278 transcription datasets in the E.coli model and more than 0.5 absolute value correlation across 17,382 RNA-seq samples in the Human model). In contrast, the majority of absolute correlations between reactions in different modules concentrated around zero supporting the presence of the inferred underlying modular structure.”

R1.3. The authors casually discuss and dismiss several concerns without describing the basis for such statements or being more specific in what they mean.  For example, on line 234 the authors state that “reduction of reversible antiporter reactions don’t affect overall model capabilities”.  How are the authors defining model capabilities? How was this determined?  We understand that they likely mean capabilities in terms of catabolic and anabolic capabilities, but what about gene essentiality or other model predictive capabilities?  The authors should be careful with their language and statements like these, and may need to demonstrate that no other model capabilities are changed based on removing such reactions.

**R:** We have clarified in the revised version what we mean with no change in the model capabilities. Moreover, we have included in supporting materials the simulations done to justify this statement. The results section was modified as follows.

“Notably, this reduction of reversible antiporter reactions does not affect the overall model capabilities in terms of metabolites that the model is able to consume and produce and maximum specific growth rate (details of simulations in Supporting file 1 and Supporting Table S5).”

R1.4. We commend the authors use of RNA transcription data to bolster their results. However, it’s difficult for us to validate these results without deposition of data (both RNA transcription data and the model simulation results).  We understand that the transcription data is from a separate published study but the outcome of the filtering steps (e.g. describe on line 199) are important to evaluate.

**R:** We have made available both the code used to process the mRNA data (Supporting file 2) and simulation results (Supporting Table S4 and S7) for both test cases.

Minor Comments:

Line 59: add “of metabolism” – reconstructions can be constructed for several other systems

**R:** Replaced for “Genome-scale metabolic reconstructions…”

Line 61 – “not”

**R:** Corrected

Line 270 “visually” may not be the correct word

**R:** Replaced for “While seemingly less profound…”

We encourage another round of grammar checks to address issues such as those above.

**R:** We thank the reviewer for the suggestions and pointing the above errors. We have corrected these in the revised article. Additionally, we have carefully reviewed the revised article for the presence of similar errors and typos.

**Reviewer #2**

The work by Martinez et al aims to identify well defined metabolic modules in genome-scale metabolic models. To this end the authors focus on metabolically connected reversible reactions that are separated by irreversible reactions form each other. Each of these reversible cluster defines one model.

The question of identifying modules in metabolism is very interesting and — in my understanding — has not been satisfactorily been answered so far. The approach by the authors is interesting. I do have, however, some concerns that relate to the strategy taken by the authors and the stability of the identified modules.

**R:** We thank the reviewer for the positive appraisal of our approach. Below, we address the reviewers’ concerns related to the general strategy for the identification and analysis of the generated modules.

Major Comments:

R2.1. The authors remove currency metabolites from the analysis. This is a key step as it is removes many links. Thus, it needs to be clearly defined what abundance makes a metabolite a currency metabolite. If the threshold that separates currency metabolites from non-currency metabolites is changed, do the cluster remain the same?

**R:** We thank the reviewer for raising this central point to our approach. Indeed, the original submission omitted any explanation about the criteria behind the choice of currency metabolites to be removed from the networks. Furthermore, we did not make explicit the list of removed metabolites. To address this limitation, we have made available the list of the reversible reactions of the models with and without currency metabolites (Supporting Tables S10 and S11). Rather than a concentration-based criterion, we employed a connectivity criterion to determine these metabolites (see Methods). We note that this topology-based criterion can be readily applied to any network as it solely rests on the structure of the network as opposed to concentration-based choices. Importantly, the removal of currency metabolites was carefully done for each reaction, so that the metabolic functions of the network were preserved. For instance, both degradation and synthesis pathways for currency metabolites were kept intact in the network as removal of the latter would inevitably lead to a loss of metabolic function. We have added in Methods an explanation about what we considered a “currency metabolite”.

“A novel approach to divide the metabolic network into functional modules (metabolic pathways) was developed. First, the currency metabolites (co-factors and moieties), defined as highly connected metabolites were removed from the network reactions. The removal of currency metabolites was done manually based on the nature of the metabolites in each reaction. In order to preserve the metabolic functions of the network, currency metabolites were kept in reactions that synthetise or degrade consume them (Supporting Tables S10 and S11).”

R2.2. Removing links (currency metabolites and anti porters) is the key for the success of the presented method. In fact previous attempts to identify modules in (genome-scale) metabolic networks that did not remove links only succeed in identifying modules for the optimal flux cone, but not for the general, see <https://doi.org/10.1007/s00285-013-0731-1> and <https://doi.org/10.1038/srep00580>. Thus, I believe that removing links needs to be more thoroughly justified. In particular the removal of anti-porters appears to me as an adhoc assumption to make the method work. Also I encourage the authors to discuss similarities and differences between the modules identified by the authors to the optimal modules identified in the mentioned articles.

**R:** We thank the reviewer for bringing this point and the additional references to our attention. We agree that the applied assumptions for removing links (by removing currency metabolites and antiporter reactions) require further explanation and justification, since these are critical for the scalability and effectiveness of our approach. We have previously addressed in our responses the limitations for the removal and selection of currency metabolites (Comment R2.1) and removal of antiporter reactions (Comment R1.3). Finally, we have included and discussed the suggested [5, 6] and other relevant references [7, 8] presenting alternative approaches for the identification of modules in metabolic networks. In the Discussion, we have included the following paragraph addressing this choice:

“The deliberate omission of energy/redox co-factors was critical for the identification of thermodynamically isolated modules. Clearly, these modules are coupled through energy and redox to other reactions, however, the coupling is to the tightly maintained global pool rather than between any two individual reactions. Arguably energy/redox homeostasis – maintaining energy charge and the ratios of various redox partners – is a more global regulatory principle (see for example [42]) than the modules identified. Conversely, assuming that coupling through energy/redox links individual reactions would speak against modularity, e.g., suggest that all gene regulation is globally coordinated with no modularity, which is clearly not accurate (operons in bacteria is a clear example of modularity). The thermodynamic isolation hereby employed focus on is the isolation achieved by irreversible reactions – commonly subject to allosteric regulation – that ensures the products have no impact on substrate concentrations or the reactions upstream of the substrates. Importantly, once modules have been indentified, currency metabolites (redox and co-factors) may be reincorporated to the respective reactions if desired. For example, a kinetic model of a module would typically include cofactors as fixed concentration external metabolites [43, 44]. It is for the sole purpose of identifying modules that currency metabolites are reversibly removed.”

R2.3. A nice feature of the work by Kelk et al (see above) is that the modules identified by Kelk et al. can nicely explain the combinatorial explosion of elementary flux modes (EFMs) in large metabolic networks, as they originate from the combination of the EFMs in the modules. Can such an argument be translated to the modules identified here as well?

**R:** Absolutely. The modules identified by our method are essentially Flux Topes (FTs) as defined in [8], and rely on the “isolation property” due to the removal of currency metabolites for their identification, as opposed to the classical enumeration of EFMs for the biomass-optimal cone [6]. In the latter case, Gerstl et al. [8] showed that there exists and exponential increase in the number of EFMs supporting larger FTs, which is consistent with the results of Kelk et al. [6] for the enumeration of EFMs for particular flux patterns of metabolic sub-networks (i.e., modules).

R2.4. With respect to the feasible reversible flux space identified by the authors, what is its relation to the reversible flux cone constraint by the set of minimal metabolic behaviors (MMB, see <https://doi.org/10.1016/j.dam.2008.06.039>). I think, they are the same. If so, what is the novelty in the proposed concept?

**R:** The Minimal Metabolic Behavior (MMB) concept relies on an outer description (non-negativity flux constraints) rather than on an inner description (non-zero reaction sets) of the flux cone. The latter is generally used to describe the solution space of metabolic networks, which is the basis for EFM-based methods for pathway analysis. Importantly, MMB seeks to determine a minimal set of *irreversible* reactions involved in a non-zero flux solution vector (which by construction will yield flux solutions with some zero entries), whereas our approach seeks to determine maximal flux solutions where all reversible reaction directionalities are fixed. As mentioned in the previous comment, DTs are fundamentally equivalent to FTs [8], albeit the scope, assumptions and calculation methods differ. For example, DTs are computed on isolated modules identified under a specific growth condition, whereas FTs are computed for the entire network under optimal biomass growth. Thus, while FTs speak of the overall coordination of reaction directions in the network, DTs represent feasible flux patterns in local subnetworks derived from removing currency metabolites.

R2.5. I do not understand if the authors assume a particular medium or if they take the models with all exchange reactions unconstrained. In the latter case, I again wonder if similar modules arise as for a specific medium. I would expect that many reversible reactions turn irreversible resulting in a much smaller topological flexibility within a fixed environment. This also touches on the validation with the expression data of 54 different human tissues. Why do the authors not use tissue specific genome-scale reconstructions for this analysis?

**R:** We have addressed the concern about the conditions for simulation in the response to Comment R1.1 and made explicit that the application of the proposed approach was under specific growth conditions (see Methods and Supporting Tables S8 and S9). Finally, we did not employ tissue-specific reconstructions as our goal was to solely use the network topology (defined by reactions directionalities) to infer metabolic modules that could be later correlated with expression data. To arrive at a context-specific model, one typically uses expression data to identify active/inactive reactions, which would have us required to use the same data twice – one for the generation of the tissue-specific model and another for the validation of the expression patterns. This would ultimately have affected the validation of the approach, and thus, we decided against using tissue-specific reconstructions.

R2.6. The authors argue that their method identifies thermodynamically isolated modules and that reversible reactions are highly independent of each other. I like to disagree that these modules are isolated and that reversible reactions are highly independent of each as thermodynamic coupling is still possible via currency metabolites. For instance, the reversible Glutamate dehydrogenase in E. coli turns into a thermodynamic bottleneck if grown on glucose, see <https://doi.org/10.1128/jb.176.15.4664-4668.1994>

**R:** As discussed above, we fully recognize that energy and redox status forms part of a higher level regulation, but the “communication” between generators and consumers is indirect through the regulated pool. We do not believe the example provided demonstrates a thermodynamic couple:

GS/GOGAT: GS is nearly irreversible and highly regulated both transcriptionally and allosterically (EcoCyc). It is part of the nitrogen two component operon and expression is high at low ammonium, while specific activity appear to be allosterically inhibited via PTMs under low glucose conditions. It is not a dramatic shift in ATP/ADP ratio that reduces its importance at high ammonium, low glucose/ATP conditions. GOGAT (GltBD) is irreversible and its expression regulated by a range of global regulators including Nac, which is activated by the nitrogen two-component system.

GDH: GDH achieves nitrogen assimilation without expending ATP. At very low levels of ammonium (μM), the reaction nears equilibrium (using eQuilibrator (<https://equilibrator.weizmann.ac.il/>) and assuming standard concentrations for other metabolites ([10.1038/nchembio.186](https://dx.doi.org/10.1038%2Fnchembio.186))). In standard culture media like M9, however, with ammonium at 20 mM, the reaction is practically irreversible.

There is no thermodynamic coupling between the two assimilation systems. Rather the more expensive GS/GOGAT is only activated when ammonia is too low for efficient GDH assimilation, the choice modulated with energy availability. This type of high level optimization happens without dramatic changes in co factor (ATP/ADP and NADPH/NADP) ratios and hence does not involve thermodynamic coupling.

R2.7. The question of how many directed topologies exist within a given environment has been theoretically addressed in terms of so called flux topes, see <https://doi.org/10.1093/bioinformatics/bty550>. I think it would be interesting to compare the authors heuristics with the exhaustive method—at least on a theoretical level.

**R:** We greatly thank the reviewer for pointing us to this work as it holds various similarities. We have discussed the latter in the Introduction and Discussion of the revised article. Below is our modification to the main text:

(Last paragraph of Discussion) ” The *E. coli* iJO1366 metabolic network flexibility was studied using the identified modules. An efficient depth-first search algorithm using simple infeasible mass balance and loopless rules was developed to explore the topological flexibility of the modules by enumerating all feasible DTs. We note that this amounts to enumerating all the flux topes in these (currency-free) subnetworks [23]. The analysis revealed only a weak coupling between structural reversible reactions in the largest module, which points to an overall high topological flexibility providing a high degree of robustness [45]. Strong coupling was only found between some boundary (exchange) and internal reactions consuming a common metabolite, which is known to be the case as exchange reactions can exert massive coupling and blocking of reactions at the boundary of metabolic networks [31].This observation is true across the modules. Assuming that modules operate independent of each other, the topological degree of freedom of the E. coli iJO1366 model was determined to be 200 (79%) out of a theoretical maximum of 248. This number represents an upper bound on the number of directionalities that must be determined to fix the topological state of the metabolic network. A more exact estimate would be obtained by enumerating all the feasible DTs in the entire network as whole, which is unfortunately impractical at this scale [23]. Still, we can conclude that except for linear pathways, reversible reactions operate practically independent of each other, granting both flexibility and robustness against internal and external perturbations [22, 34, 46, 47].

R2.8. The authors develop a heuristics to compute directed topologies an suggest that with their algorithm it is likely to find all feasible directed topologies. I think this statement needs to be explicitly shown. Either in a smaller network, like Ecoli_core, <http://www.asmscience.org/content/journal/ecosalplus/10.1128/ecosalplus.10.2.1#backarticlefulltext>, or ECC2, <https://doi.org/10.1038/srep39647>, or by restricting the analysis to the optimal flux cone in a large network.

**R:** Our method to compute the feasible directed topologies consists in a depth-first search where the infeasible DTs are rejected based in infeasible flux patterns previously determined based on loopless and mass balance rules. Some extra missing infeasible patterns were created by a heuristic approach. For all but the largest module, a brute force method was used to exhaustively enumerate all feasible DTs. We found that for all those modules our method produced the same number of DTs. This is equivalent to demonstrating the approach in smaller networks as suggested above. Only in the case of the largest module could this verification not be performed due to the excessive computation time required. However, we did the same comparison using a large sample of DTs and found that the percentage of feasible DTs was consistent between both methods.

**Reviewer #3**

The work by Martínez et al focuses on the well-known problem (re)interpretation of metabolic networks, finding modules. This work focuses on thermodynamic perspective, reversibility of reactions and topological properties of some (sub) networks. The novelty of the proposed method is questionable, if at all unclear.

The idea seems interesting at first yet there are several points to be addressed in this paper, in particular the novelty is questionable and the criteria is rather poorly described and is unclear what is the difference between earlier and proposed concepts. The following points need to be addressed before any decision.

**R:** Below, we have addressed the reviewer’s concerns about the novelty and underpinning assumptions of our approach. We acknowledge that some key references to previous works were missing in our initial submission. We have now included and discussed these in the revised article version. Moreover, we have better explained the criteria and justification for the removal of currency metabolites, which is key and distinctive of our approach. Overall, the revised version highlights the novelty and differences with previous approaches, and clarifies the general methodology.

Major Comments:

R3.1. P16, L279. The authors quantify the flexibility by (number of?) possible directed topologies. The authors need to elaborate this criterium, as this already has been tackled by the elementary modes concept. How this metric is different than earlier defined concepts?

**R:** We thank the reviewer for this point as we acknowledge it was not clear in the original submission. We quantify the topological flexibility of metabolic networks by the number of feasible DTs composing them. Notably, DTs are very similar to the Flux Topes (FTs), which has been recently applied for exploring the flexibility of biomass-optimal network states [8]. We note that in both cases maximal pathways are computed with all the reactions carrying flux. This strategy is fundamentally different from EFMs, in that: 1) DTs/FTs are not elementary, and thus, they are not EFMs (although DTs/FTs can be decomposed into EFMs [8]), and 2) EFMs cannot be exhaustively enumerated in practice for large models due to combinatorial explosion, whereas DTs/FTs can be under some assumptions. Furthermore, DTs and FTs are more appropriate for the analysis of metabolic flexibility as they represent a unique and (in theoretical terms) consistent [12] set of (sub)networks that are more representative of the coordination of reaction directions, and thus, topological flexibility [8]. Finally, a key difference between DTs and FTs is that our approach employs an optimization-free method that enables exhaustive enumeration of all the DTs in isolated modules identified under a specific (growth) condition (by means of removal of currency metabolites), whereas FTs are computed using a reverse search (optimization-based) algorithm under optimal growth conditions for the entire network. Naturally, the latter scales worse and is not useful for identifying metabolic modules. We have included some of these points in the Discussion of the revised article (refer to Comment R2.7).

R3.2. When the currency metabolites are removed from the system, what happens to the reactions that consumes/produces these? The reason to focus on this is as follows: the presence of internal loops or closed cycles can be drawn from the analysis of the stoichiometric matrix (e.g. analysis of null space as already mentioned in the manuscript) which is somewhat “distorted” by the removal of these currency metabolites.

**R:** We thank the reviewer for raising this point. This was an omission in the first submission. In the revised article, we have made explicit that the loop law matrix is constructed using the original reaction stoichiometries, thereby avoiding any distortion of the generated loop laws (see last paragraph of the Methods).

R3.3. Focusing on finding the properties of the sub-network, how the proposed method is different from elementary flux modes (or extreme pathways) problem is not clear, and judging from enumeration approach accompanied by the classical analysis of stoichiometric matrix, it seems that this is study a minor contribution to the field.

**R:** We have addressed the concern about the difference and benefits of DTs for the analysis of network flexibility in Comment R3.1. In regards to the enumeration algorithm, to the best of our knowledge, previous pathway analysis (i.e., EFM-based) methods for module identification require – at some point – the solution of optimization problems [5-7], which severely hinders their applicability in larger models. In contrast, our approach relies on the definition of *infeasible* flux patterns, which can be readily checked in any (sub)network without the need to solve an optimization problem. Importantly, while our approach does not guarantee finding all possible DTs (up to 10^11^), in practice, it did find them all whenever they could be verified (refer to Results).

**References**

1.	Martínez VS, Quek LE, Nielsen LK. Network Thermodynamic Curation of Human and Yeast Genome-Scale Metabolic Models. Biophysical Journal. 2014;107(2):493-503. doi: DOI 10.1016/j.bpj.2014.05.029. PubMed PMID: ISI:000339148500025.

2.	Martínez VS, Nielsen LK. NExT: Integration of Thermodynamic Constraints and Metabolomics Data into a Metabolic Network. Metabolic Flux Analysis: Springer; 2014. p. 65-78.

3.	Acuña V, Marchetti-Spaccamela A, Sagot M-F, Stougie L. A note on the complexity of finding and enumerating elementary modes. Biosystems. 2010;99(3):210-4. doi: <https://doi.org/10.1016/j.biosystems.2009.11.004>.

4.	Acuña V, Milreu PV, Cottret L, Marchetti-Spaccamela A, Stougie L, Sagot M-F. Algorithms and complexity of enumerating minimal precursor sets in genome-wide metabolic networks. Bioinformatics. 2012;28(19):2474-83. doi: 10.1093/bioinformatics/bts423.

5.	Müller AC, Bockmayr A. Flux modules in metabolic networks. Journal of Mathematical Biology. 2014;69(5):1151-79. doi: 10.1007/s00285-013-0731-1.

6.	Kelk SM, Olivier BG, Stougie L, Bruggeman FJ. Optimal flux spaces of genome-scale stoichiometric models are determined by a few subnetworks. Sci Rep-Uk. 2012;2(1):580. doi: 10.1038/srep00580.

7.	Larhlimi A, Bockmayr A. A new constraint-based description of the steady-state flux cone of metabolic networks. Discrete Applied Mathematics. 2009;157(10):2257-66. doi: <https://doi.org/10.1016/j.dam.2008.06.039>.

8.	Gerstl MP, Müller S, Regensburger G, Zanghellini J. Flux tope analysis: studying the coordination of reaction directions in metabolic networks. Bioinformatics. 2019;35(2):266-73. doi: 10.1093/bioinformatics/bty550.

9.	Valgepea K, de Souza Pinto Lemgruber R, Meaghan K, Palfreyman RW, Abdalla T, Heijstra BD, et al. Maintenance of ATP Homeostasis Triggers Metabolic Shifts in Gas-Fermenting Acetogens. Cell Systems. 2017;4(5):505-15.e5. doi: 10.1016/j.cels.2017.04.008.

10.	Saa PA, Nielsen LK. Construction of feasible and accurate kinetic models of metabolism: A Bayesian approach. Sci Rep-Uk. 2016;6:29635. doi: Artn 29635

10.1038/Srep29635. PubMed PMID: WOS:000379724100001.

11.	Kozaeva E, Volkova S, Matos MRA, Mezzina MP, Wulff T, Volke DC, et al. Model-guided dynamic control of essential metabolic nodes boosts acetyl-coenzyme A–dependent bioproduction in rewired Pseudomonas putida. Metab Eng. 2021;67:373-86. doi: <https://doi.org/10.1016/j.ymben.2021.07.014>.

12.	Acuña V, Chierichetti F, Lacroix V, Marchetti-Spaccamela A, Sagot MF, Stougie L. Modes and cuts in metabolic networks: complexity and algorithms. Biosystems. 2009;95(1):51-60. Epub 2008/08/30. doi: 10.1016/j.biosystems.2008.06.015. PubMed PMID: 18722501.
