# Supplementary material for: The topology of genome-scale metabolic reconstructions unravels independent modules and high network flexibility
Source: PLoS Comput Biol. 2022 Jun 27;18(6):e1010203. doi: 10.1371/journal.pcbi.1010203 (PMC9269948; doi:10.1371/journal.pcbi.1010203)
Supplement: Attachment — Submitted filename: Response letter 4.5.22.docx [file pcbi.1010203.s007.docx]

**Response to Reviewers' comments**

**PCOMPBIOL-D-21-01428R1**

**The topology of genome-scale metabolic reconstructions unravels independent modules and high network flexibility**

**Due Date:** May 08, 2022

**Reviewer #1:** The authors have satisfactorily addressed all of our comments

**Reviewer #2:** Thank you, authors, for your detailed response and the modifications to your manuscript.

My concerns have been sufficiently addressed.

I still wonder about the definition of cofactors and the impact of this definition on your results. Especially, I regret that you decided not to test the sensitivity of your results on a slightly different definition of cofactors (e.g. by increasing or decreasing the number of required links that make a metabolite a cofactor).

At the least I'd ask you to make your current statement more precise "defined as highly connected metabolites were removed from the network reactions. The removal of currency metabolites was done manually based on the nature of the metabolites in each reaction. In order to preserve the metabolic functions of the network, currency metabolites were kept in reactions that synthesize or degrade consume them (Supporting Tables S10 and S11)." Please could you include the minimum number of links that makes a metabolite removable, (and any additional guiding principles that were used in your manual curation) For instance, in case there are 2 alternative synthesis pathways, did you keep both in or just one? etc.

**R:** In order to address this concern, we have included a more detailed explanation about the selection of currency metabolites for removal in this analysis. Furthermore, a supplementary table was included with the list of currency metabolites removed. The main text was modified to:

“First, the currency metabolites (co-factors and moieties), defined as highly connected metabolites with a carrier function (e.g., electrons and chemical groups), were removed from the network reactions. The removal of currency metabolites was done manually based on their role in each reaction. For instance, if a currency metabolite was participating as carrier in the reaction, it was removed, otherwise it was kept (refer to Supporting Table S10 for the complete list of removed metabolites). In addition, if it was not clear which metabolites were the co-factors and main substrates/products of the reaction (e.g., K+-Cl- cotransport: cl[e]+k[e]=cl[c]+k[c]), all metabolites were kept. In order to preserve the metabolic functions of the network, currency metabolites were kept in all reactions that synthetize or degrade them, as well as when a metabolite was being used as a building block (e.g., Acetoacetyl-CoA:acetate CoA-transferase: acac+coa=aacoa) (Supporting Tables S11 and S12).”

As for your ability on "isolated thermodynamic modules" I remain skeptical. Particularly I disagree with your response that "There is no thermodynamic coupling between the two assimilation systems" with respect to GS/GOGAT. Previously, we found that there is a coupling transmitted via cofactors, see <https://doi.org/10.1038/srep08930>. I'd love to discuss these aspects with you in future. However, this disagreement does not hinder publication.

**R:** We will also be delighted to discuss this issue with the reviewer in more depth on another occasion.

**Reviewer #3:** The revised work by Martínez et al focuses on determination of directed topologies, finding modules from thermodynamics perspective. There are several major changes in the article and the new version is clearly improved version.

My earlier comments were centered on three points:
- metrics in enumerating different modules
- difference with earlier work
- fate of currency metabolites

These points (fate of currency metabolites to a much lesser extent) have been addressed by the authors.

Finally, the use of log2 in determining the degree of freedom needs to be motivated.

**R:** We are pleased that the reviewer appreciated the improvements made to the manuscript. In regards to the selection of log2 for measuring the topological degrees of freedom, this captures the fact that reversible reactions have two possible directionalities (forward or reverse), increasing geometrically (base 2) the total number of possible topologies with increasing number of reversible reactions. If we denote by N the number of feasible DTs, then log2(N) describes the number of reversible reactions that are still “free” and represent the minimum number of reversible reactions that must be directionally fixed in order to specify a distinct feasible DT. The motivation for the use of Log2 can be found in the results section on page 13:

“We defined the "topological" degree of freedom (DoF) (Log2N) of the metabolic network (N being the total number of feasible DTs), as the minimum number of reversible reactions that must be directionally fixed in order to fix metabolism to a distinct feasible DT. From a practical point of view, it may be possible to identify the metabolic state of the cell under certain growth conditions by determining the direction of key reactions by the use of metabolomics data and thermodynamic principles [32, 33, 37-39], if the “topological” DoF is a relatively small number.”
